# Primed to overreact: virus-induced IFN contributes to antibody-mediated anaphylaxis

**DOI:** 10.1172/JCI203729

**Published:** 2026-02-16

**Authors:** Pablo Penaloza-MacMaster

Type I IFNs (IFN-I) are upregulated upon viral infection to restrict viral replication and orchestrate innate and adaptive immune responses. Yet, a growing body of literature has revealed that IFN-I can also have deleterious consequences, particularly when dysregulated or excessive (1–4). Clinical observations have suggested that among these consequences is an increased susceptibility to anaphylaxis during viral infections, with speculation that virus-induced IFN-I may be implicated, although the underlying mechanisms have remained unclear (5, 6).

In this issue of the *JCI*, Elwy, Abdelrahman, and co-authors (7) examined the effects of virus-induced IFN-I in mouse models of anaphylaxis and demonstrated that IFN-I can exacerbate antibody-mediated hypersensitivity reactions. Rather than directly mediating anaphylaxis, IFN-I induced upregulation of Fc receptors on innate immune cells, lowering the threshold for catastrophic anaphylaxis driven by antibody effector functions. Using various models of viral infection in mice, this study showed that IgG-mediated anaphylaxis was amplified during acute viral infection, driven predominantly by the Fc receptor FcγRIV, a murine homolog of human FcγRIII. Mechanistically, viral infection induced IFN-I responses, which then upregulated FcγRIV expression on inflammatory monocytes, rendering the animals more susceptible to antibody-mediated anaphylaxis. Blockade of IFN-I signaling abrogated FcγRIV upregulation and protected infected mice from anaphylaxis, establishing IFN-I as an essential upstream pathway implicated in the disease. In addition, Elwy, Abdelrahman, and co-authors showed that FcγRIV engagement on inflammatory monocytes triggered the production of platelet-activating factor (PAF), a potent vasoactive mediator responsible for lethal anaphylaxis. Pharmacologic blockade of the PAF receptor rescued the mice from anaphylaxis, highlighting a druggable downstream pathway. The authors also extended some of these findings to humans by demonstrating increased frequencies of FcγRIII-expressing monocytes in patients with COVID-19, who are known to exhibit a predisposition to allergic reactions (5, 6).

Together, these findings show that virus-induced IFN-I can act as a double-edged sword that, despite its role in coordinating antiviral defenses, can also predispose the host to lethal anaphylaxis. This work adds an important dimension to the IFN-I paradox and identifies Fc receptors and PAF as potential therapeutic targets to prevent anaphylaxis associated with viral infections.

## Version 1 02/16/2026

Electronic publication
